# Stabilization of refractive error and associated factors following small incision phacoemulsification cataract surgery

**DOI:** 10.1186/s12886-021-02221-w

**Published:** 2022-01-06

**Authors:** Ammar M Khan, Derek M. Waldner, Micah Luong, Emi Sanders, Andrew C. S. Crichton, Bryce A. Ford

**Affiliations:** 1 Division of Ophthalmology, University of Calgary, 49 Richard Way SW, Calgary, AB T3E 7M8 Canada; 2 Cumming School of Medicine, University of Calgary, Calgary, Canada

## Abstract

**Background:**

Accumulating evidence suggests that refractive stabilization occurs rapidly following small incision cataract surgery. Nonetheless, many guidelines still suggest waiting four to 6 weeks before prescribing corrective lenses. This study was undertaken to supplement the existing literature regarding refractive stabilization, and evaluate multiple contributing factors that could dissuade clinicians from confidently correcting refractive error in the early post-operative course following routine cataract surgeries.

**Methods:**

Adult patients undergoing phacoemulsification cataract surgery with uncomplicated surgeries and post-surgical courses at the Calgary Ophthalmology Centre (Calgary, Alberta, Canada) were included in this prospective observational case series. Exclusion criteria included known corneal dystrophies, infectious keratitis, complicated surgery or toric/multifocal IOLs. Data was collected at weekly intervals for a total of 6 weeks. Collected data included autorefraction, visual acuity, corneal pachymetry, and effective lens position.

**Results:**

One hundred six eyes of 104 patients were included in this study. Post-operative sphere, cylinder and spherical equivalent were not significantly different at any post-operative week compared with week six, and 80–86% of patients were within 0.5D of last follow-up spherical equivalent at any week. The secondary outcomes of central corneal thickness, effective lens position and visual acuity did, however, exhibit significant differences between early post-operative weeks and last follow-up values.

**Conclusions:**

These data suggest that refractive error can be effectively measured and corrected as early as one-week post-operatively in the majority of patients, though other measures of post-operative stability including central corneal thickness, effective lens position and visual acuity can require up to 4 weeks to stabilize. Thus a conservative and pragmatic approach may be to wait until 4 weeks post-operatively prior to obtaining refractive correction following uncomplicated phacoemulsification cataract surgery.

**Supplementary Information:**

The online version contains supplementary material available at 10.1186/s12886-021-02221-w.

## Introduction

Cataract surgery is not only the definitive management for vision-obscuring cataracts, but also can correct lifelong refractive errors in myopic, hyperopic or astigmatic patients [1]. This change in refractive status of the eye is managed by meticulous pre-operative planning to determine the appropriate intraocular lens for each patient, depending on factors including axial length of the eye, corneal topography and keratometry, patient preferences and more [2, 3]. Due to the inability of artificial intraocular lenses (IOLs) to accommodate, most post-operative patients still require post-operative refractive correction either for near or distant visualization (depending on their pre-selected preferences) which can delay return to driving and other distance vision-requiring activities. Some guidelines recommend delaying refractive correction until four to 6 weeks post-operatively [4], likely based on previous large-incision iterations of cataract surgeries, which could require substantial post-surgical healing [5, 6]. More recent evidence, however, suggests that with small incision techniques, post-surgical time to refractive stabilization might be substantially less – even as low as 1 week [7–10]. Given the reduced quality of life associated with ametropia, definitive determination of the optimal post-operative period at which patients can receive correction without risk of further refractive changes is warranted [11]. Additionally, it has been shown that definitive post-operative refractive error measurements improve second-eye outcomes for patients with sequential cataract surgeries (especially for patients desiring mono- or mini-monovision) [12]. Finally, burgeoning technology in IOLs - including RxSight’s RxLAL light-adjustable lens - offer the potential for post-operative refractive correction, but require a definitive, stable, post-surgical refractive measurement prior to any adjustments [13]. Thus, increasing confidence in early post-surgical refractive results will allow ophthalmological surgeons to improved outcomes for patients without burdening them with excessive intervals between surgeries, or delayed return to emmetropic correction.

This study was undertaken to supplement the literature on time to refractive stabilization following small-incision phacoemulsification cataract surgery, with additional parameters potentially associated with refractive instability evaluated also, including cylindrical axis, visual acuity (VA), central corneal thickness (CCT), and effective lens position (ELP). If congruent with previous recent reports on post-surgical stabilization, these data will provide additional evidence for early correction of refractive error following this common surgical technique and contribute novel data on factors associated with refractive stability.

## Methods

This was a prospective clinical study of patients undergoing cataract surgery recruited between September 2015 and end of April 2019 by two ophthalmic surgeons (A.C. and B.F.) in Calgary, AB, Canada. Inclusion criteria for the study included age over 17 years of age and undergoing cataract surgery in one or both eyes with a one-piece hydrophobic acrylic intraocular lens. Exclusion criteria included age 17 and below, infectious keratitis, corneal dystrophies, multifocal/EDOF (extended depth of focus) or toric IOLs, and inability to provide informed consent or attend all postoperative visits. This study was approved by the University of Calgary Conjoint Health Research Ethics Boards (CHREB) under protocol number REB-15-1745 and was in accordance with the Declaration of Helsinki and Good Clinical Practice guidelines.

Cataract surgery was performed under topical anesthetic using a clear corneal 2.2 mm temporal incision followed by phacoemulsification and intracapsular insertion of intraocular lens (IOL). Pre-operative measurements were performed with the IOLMaster700 (Carl Zeiss Meditec, Jena, Germany). Post-operative care included a routine schedule of topical antibiotics, non-steroidal anti-inflammatories, and steroid drops to the post-operative eye, with minor variations on tapering pending patient characteristics determined by the surgeon. Following surgery, patients returned for weekly assessments for 6 weeks (± 3 days of scheduled weekly follow-up). At each visit, collected patient data included qualitative assessment for surgical complications, as well as visual acuity testing, corneal pachymetry (Pachmate DGH 55; DGH Technologies, Exton, PA, USA), keratometry and automated refraction (Tomey RT-7000; Tomey USA, Phoenix, AZ, USA). Single data points for each measurement were collected at each visit.

The primary outcome measures were refraction error (sphere and cylinder) and refractive stability, defined as spherical equivalent +/− 0.50 dioptres (D) of the last follow-up value (week five or six). Secondary outcome measures were uncorrected visual acuity, central corneal thickness (CCT), effective lens position (ELP) and cylindrical axis for patients with >1D of astigmatism. If patient data was absent for weeks 5 and 6, these data sets were excluded from comparisons to last follow-up and analyses of stability, but not analyses of individual weeks. No other data was excluded from analysis. Visual acuities were transformed from Snellen to LogMAR by the method outlined in Tiew et al. [14] Effective lens position (ELP) was measured as anterior chamber depth (ACD) after insertion of IOL, as previously reported [15]. Statistical analysis and figure generation was performed with Prism GraphPad v8.0. Weekly spherical equivalent, sphere, cylinder, cylindrical axis, visual acuity, CCT and ELP values were evaluated for significant differences by repeated measures one-way ANOVA and Dunnett’s multiple comparisons test.

Logistical regression was used to evaluate age, sex, eye, glaucoma, number of medications, change in effective lens position (week one to last follow-up) and change in central corneal thickness (week one to last follow-up) for correlations with spherical equivalent stability across the study period.

## Results

### Patient demographics

One hundred six eyes of 104 patients were included in the study, with no eligible patients excluded for post-operative surgical complications. Two eligible patients withdrew consent to participate after beginning the study. Of the 104 patients, three were lost to follow-up prior to week five, and thus were excluded from stability and comparative analyses. Cumulative data collection was 92.1% with losses attributed to missed appointments and occasional equipment malfunctions. Demographic information of patients included in this study can be found below in Table 1.

**Table 1:** Demographics of patients included in this study

Demographics	
***n*=**	106
**Age (Mean ± SD)**	73.2 ± 7.7
**Male [n (%)]**	43 (40.1%)
**OD Eye [n (%)]**	56 (52.8%)
**Glaucoma? [n (%)]**	32 (30.2%)
**If Glaucoma, # Meds (Mean ± SD)**	0.46 ± 0.90

### Refraction and refractive stability

**Fig. 1:**
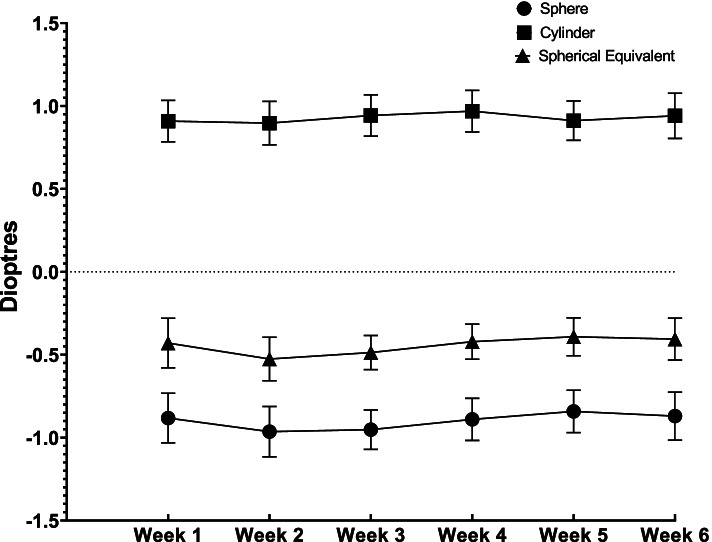
Mean ± 95% confidence intervals for sphere, cylinder and spherical equivalent measurements between one to 6 weeks post-phacoemulsification cataract surgery. Dunnett’s multiple comparison’s test revealed no significant differences between week one to five and week six means for any refractive measurements

Post-operative sphere, cylinder and spherical equivalent were not significantly different at any post-operative week compared with week six (See Fig. 1**)**. Across the study period, weekly mean values were between − 0.96 – -0.81 D, + 0.90 − + 0.97 D, and − 0.53 – − 0.39 D for sphere, cylinder and spherical equivalent, respectively. These values are consistent with the mildly myopic post-surgical refractive target planned for the majority of patients. Mean, standard deviation and *p*-values for these outcomes compared to week 6 for weekly measurements can be found in Supplementary Table 1.

**Fig. 2:**
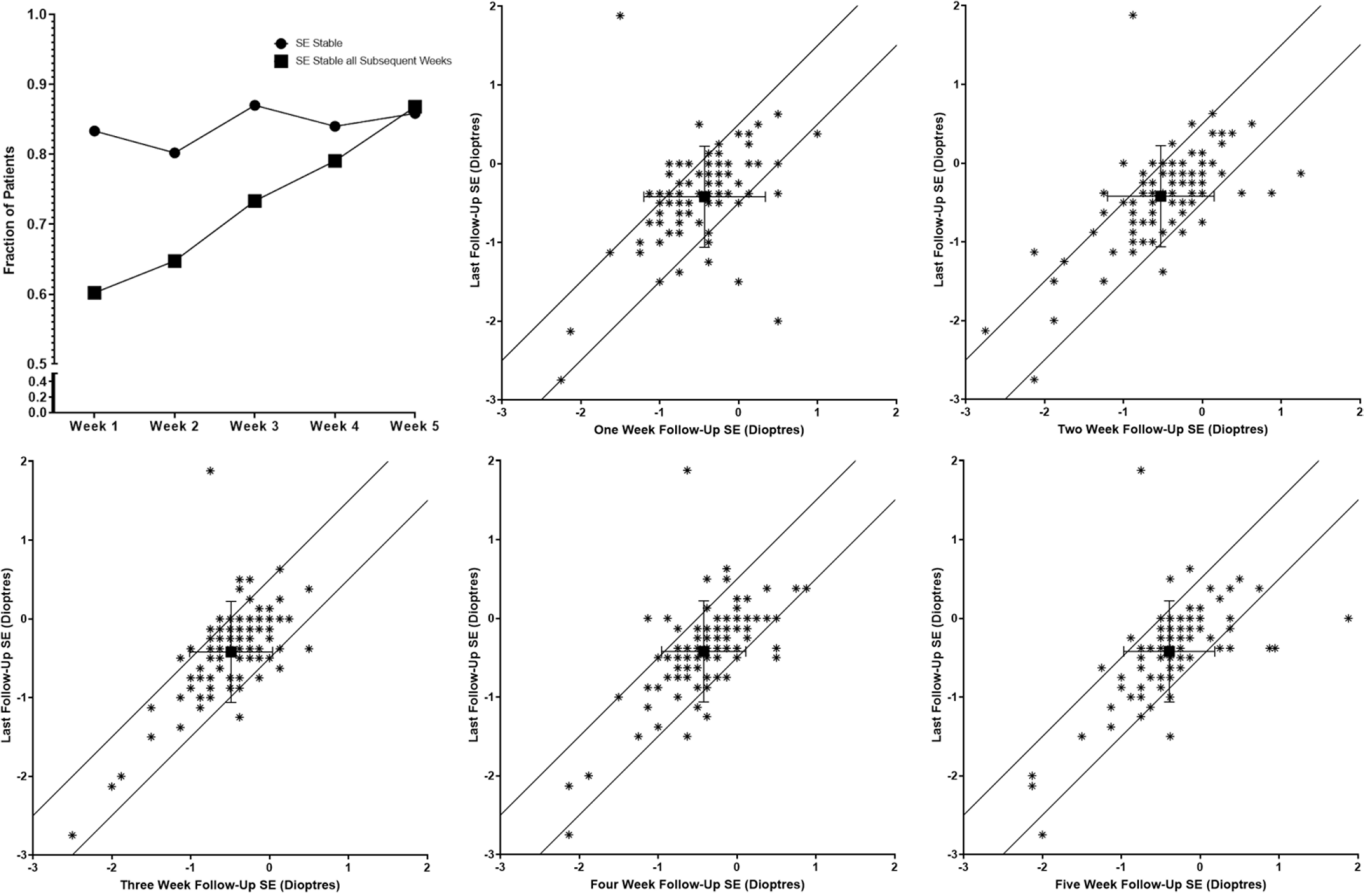
**A** Fraction of patients at each post-operative week with a spherical equivalent within 0.5 dioptres of the last follow-up spherical equivalent (circles). Squares denote the fraction of patients at each week with spherical equivalents within 0.5 dioptres that week, and all sequential weeks (i.e. all weeks for week 1, weeks 2–5 for week 2, etc.). **B**-**E** – Comparison of weekly spherical equivalent values with last follow-up value for each week. Diagonal lines demark border of stability criteria. Overlapping points appear as one. 3–7 points are outside the axis limits for the graphs

80.2–87.0% of patients met the refractive stability criteria (±0.5 D spherical equivalent of last follow-up value) at each week measured. However, this ~ 80% represented a different population at each follow-up interval, such that almost all surgical eyes met stability criteria at at least one of their follow-ups (103/106, 97.2%). 60.2% of eyes recorded a spherical equivalent within 0.5 D of their week 6 value at all post-operative visits included in this study whereas 91.5% of patients were within 1 D at all visits (See Fig. 2 and Table 2).

**Table 2:** Portions of patients within specified range of last follow-up spherical equivalent (SE) at post-operative weeks one through five

	Week One	Week Two	Week Three	Week Four	Week Five
***n*=**	102	101	100	99	99
**SE Within 0.5 D (n [%])**	85 (83.3%)	81 (80.2%)	87 (87.0%)	84 (84.8%)	85 (85.9%)
**SE Within 0.75 D (n [%])**	93 (91.2%)	91 (90.1%)	95 (95.0%)	89 (89.9%)	92 (92.9%)
**SE Within 1.0 D (n [%])**	97 (95.1%)	98 (97.0%)	99 (99.0%)	95 (96.0%)	93 (93.9%)

### Visual acuity, central corneal thickness, effective Lens position, and cylindrical Axis

**Fig. 3:**
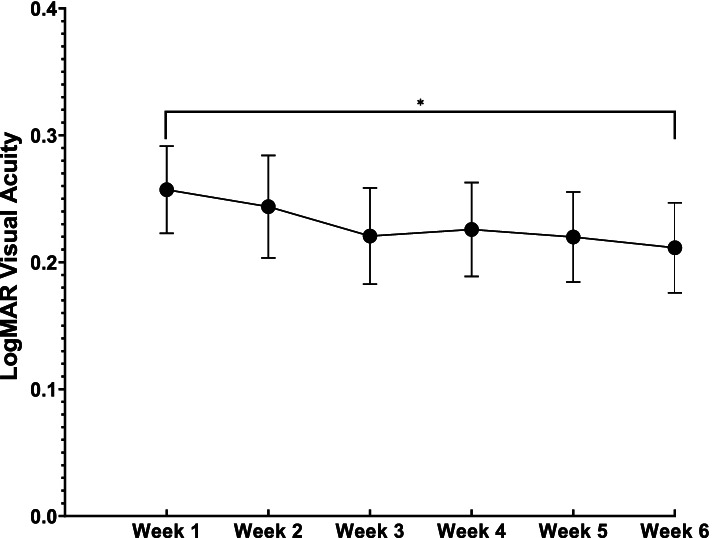
Mean logMAR visual acuity ± standard deviation measurements between one to six weeks post-phacoemulsification cataract surgery. Week one and week six means were significantly different by Dunnett’s multiple comparison test

A number of secondary outcomes were additionally evaluated for possible contributions to refractive instability. The first of these was visual acuity, recorded on via Snellen chart, and converted to LogMAR in accordance with Tiew et al. Mean logMAR visual acuity at post-operative week one was 0.26 ± 0.18 (~ 20/40 + 2), and improved by week six to 0.21 ± 0.18 (~ 20/30–2). While statistically significant, these data represent a mean improvement of only single additonal letter on Snellen visual acuity testing. There was no significant difference between any other week and week six visual acuity measurements (See Fig. 3).

**Fig. 4:**
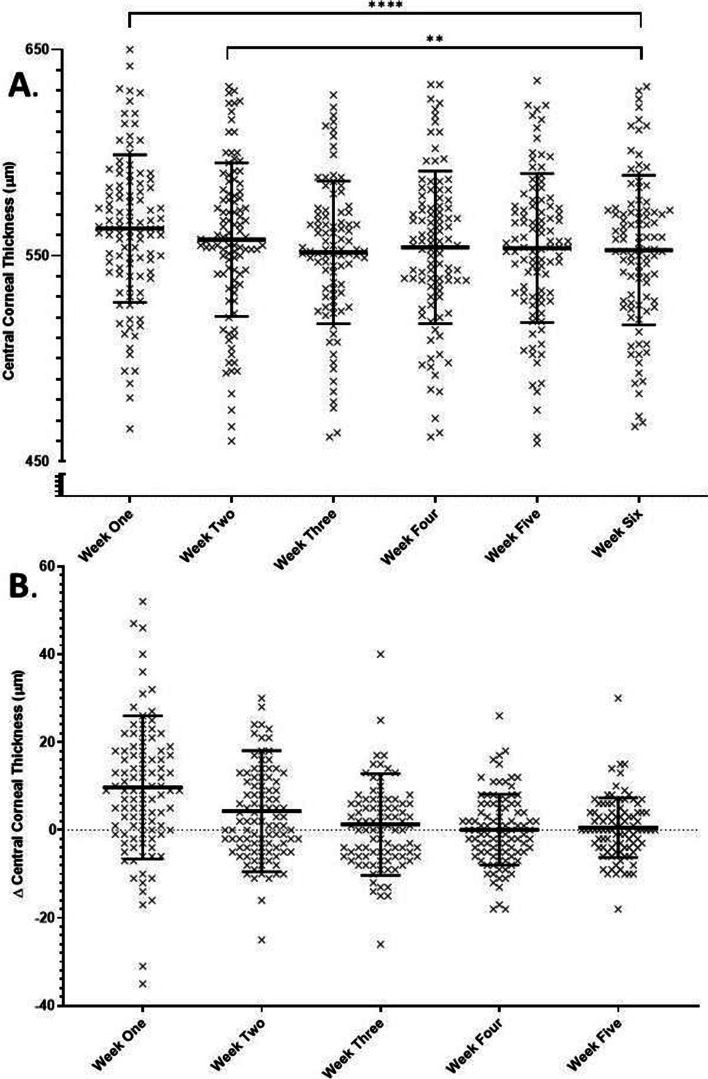
**A** Patient post-operative central corneal thickness values. There is a significant difference between weeks one and two and week six values. Line and bars indicate mean ± standard deviation **B.** Change in central corneal thickness between weekly and last-follow up values. Line and bars indicate mean ± standard deviation. Three data points are outside the axis limits

Pachymetry was also performed at each post-operative visit to evaluate central corneal thickness (CCT). CCT decreased from a mean value of 563.1 ± 35.8 μm at week one to a mean low of 551.6 ± 34.6 at week three. Mean CCT was significantly higher at weeks one (*p* = < 0.0001) and two (*p* = 0.0028) than the week six mean (552.7 ± 36.3 μm). There was no significant difference between CCT at last-follow up and any of weeks three through five (See Fig. 4).

**Fig. 5:**
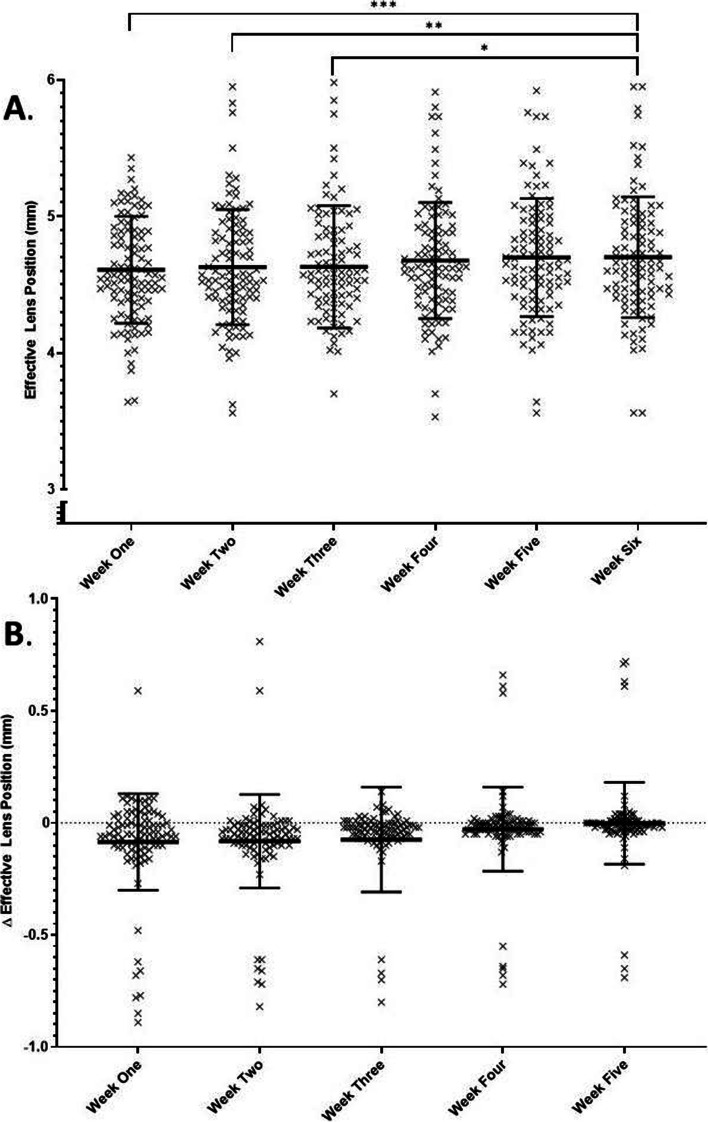
**A** Patient post-operative effective lens position values. There is a significant difference between weeks one, two three and week six values. Line and bars indicate mean ± standard deviation **B.** Change in effective lens position between weekly and last-follow up values. Line and bars indicate mean ± standard deviation. Three data points are outside the axis limits

Post-operative effective lens position (ELP) was also evaluated. Effective lens position increased from a mean value of 4.61 ± 0.39 mm at week one to a week six average of 4.70 ± 0.44 μm. Mean ELP was significantly less than the week six mean at weeks one (*p* = 0.0002), two (*p* = 0.0037) and three (*p* = 0.018). There was no significant difference between ELP at last-follow up and week 4 or 5 (See Fig. 5).

**Fig. 6:**
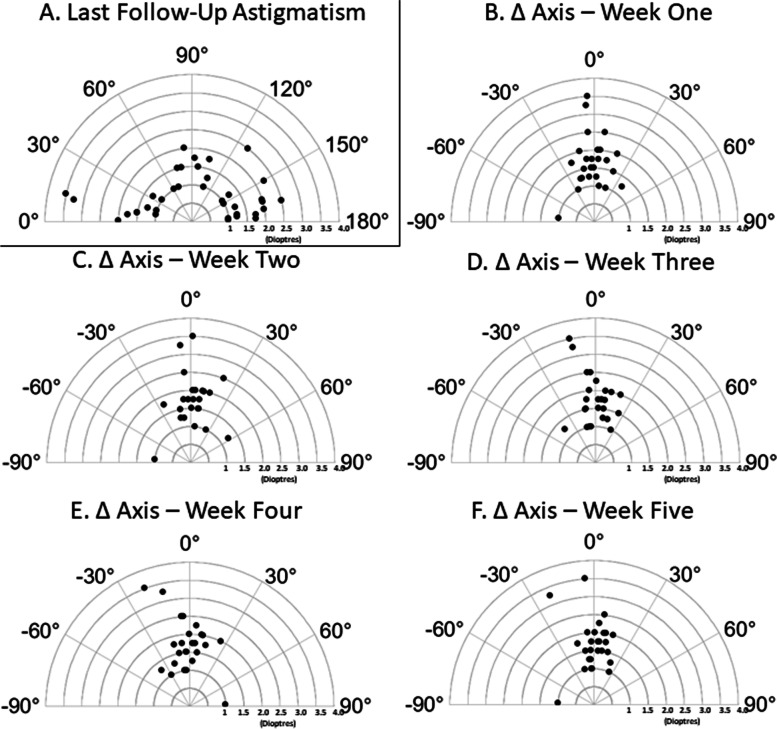
A – Autorefraction cylinder and axis values at last follow-up for astigmatic patients (> 1 dioptre cylinder). B-F – Change in cylinder axis between weekly and last-follow up post-surgical values for astigmatic patients with cylinder > 1 dioptre. Tukey’s multiple comparison’s test revealed no significant differences of cylindrical axes between any post-operative weeks

Finally, cylindrical axis was evaluated for change in patients with astigmatism >1D at both week one and last follow-up measurements (*n* = 47). Among this cohort, the last follow-up mean cylindrical refractive error was 1.59 ± 0.60 with an expected distribution of cylindrical axes. There was no significant difference between cylindrical axes between any two weeks (including last follow-up), as would be expected if there was not a post-operative change during the study. The vast majority of axis measurements were within 30^o^ of the last-follow value (Fig. 6, A) at all study weeks (Fig. 6, B-F).

The above demographic and secondary outcome data were subsequently utilized in logistical regression analysis to investigate possible correlations with refractive stability by comparing the “stable at all weeks” (spherical refractive error ± 0.5 D of last follow-up value during all weeks; *n* = 62, 60.2%) and “not always stable” (spherical refractive error < or > 0.5 D of last follow-up value at one or more visits; *n* = 41, 39.8%) groups. No significant correlation with stability was found when evaluating age, sex, eye, glaucoma status, number of medications (glaucoma cohort), change in effective lens position (week one to last follow-up) and change in central corneal thickness (week one to last follow-up).

## Discussion

The refractive stabilization of patients undergoing phacoemulsification surgery is an important question given the prevalence of cataract surgery combined with the patients desire to return to normal activities. This study attempted to answer some of these questions, of course with limitations and consideration required for clinical and practical relevance.

The mean refractive error of the study cohort (as measured by sphere, cylinder and spherical equivalent) was not significantly different between any 2 weeks, with similar standard deviation in measurements at each week. This suggests that – on average – the patient population was refractively stable at 1 week and remained so throughout the study duration. These data are consistent with previous reports showing early mean refractive stability in study populations [7–10, 16, 17].

The probability of any single patient being within 0.5 D of their last follow-up spherical equivalent ranged from 80.2–87.0% between weeks one and five, with a linear increase in the fraction of patients that remained stable all subsequent weeks beginning at ~ 60% week one. This could suggest a subset of patients that are late to stabilize, but further analysis revealed that the “unstable” cohort represented a different subset of patients at each week, such that all but 3 eyes were stable during at least 1 week prior to last follow-up despite 14.1% of patients being “unstable” at week 5. Further, logistical regression revealed no variables that had significant correlation with patient stability at all weeks. Collectively, these data suggests that the inconsistency between mean cohort refractive stability and individual patient refractive stability is most likely due to error in the autorefractor measurements of sphere and cylinder. These are device dependent, but have been shown to have significant within and between subject variability with other autorefractor devices [18, 19]. Given the study design, individual measurement errors can have a disproportionate effect on the primary outcome, especially with the last follow-up value. For example, as observed in Fig. 2, a single week 6 measurement (likely attributable to error) has resulted in “unstable” data point at weeks 1–5 (at x ≈ − 1, y ≈ 2). Thus it is likely that almost all patients are refractively stable by our defined criteria by one-week follow-up assessment. In future studies, multiple averaged measurements of the ultimate refractive error at last visit or subsequent follow up may help minimize this error.

The secondary outcomes of central corneal thickness (CCT), effective lens position (ELP) and visual acuity (VA) did, however, exhibit significant differences between early post-operative weeks and last follow-up values. CCT was significantly increased at weeks one and two, which can be clinically attributed to the post-operative corneal edema, expected following anterior segment surgery. This is in keeping with previous reports, which have shown statistically significant post-surgical corneal edema lasting days to weeks [10, 20, 21]**.** In keeping with this, VA was also significantly worse at week one compared with last follow-up (though only slightly), and trends towards stability by week three, when corneal edema appears to have resolved in most patients. ELP, by contrast, increased from a mean value of 4.61 ± 0.39 mm at week one to a week six average of 4.70 ± 0.44 mm with a significant difference in mean measurements compared to last follow-up persisting until week 4. This slight increase is to be expected as the intraocular lens settles into the capsular bag and is consistent with previous reports [22–26]**.** Given the small amplitude of movement, however, this change would not be associated with a significant change in the refractive system. Finally, the cylindrical axis in astigmatic patients (defined as having cylinder >1D at last follow-up) showed no significant changes throughout the duration of the study [27]**.**

There are several limitations of the study and study design, which limit the inferences that may be made from these data. The first of these is the error introduced by reliance on individual auto-refractor measurements (especially last follow-up as the comparative “gold standard” for each data point), as discussed above. The patient population was also devoid of possible confounding conditions or complications, such as corneal dystrophies, significant post-surgical corneal edema, or significant post-surgical anterior chamber inflammation which may effect time to refractive stability. Further, some patient demographic data that may have influenced time to stability, including prior refractive surgeries (LASIK, RK, PRK) were not collected or analyzed [28, 29]. The sub-analysis of astigmatics was also likely confounded by exclusion of toric lenses, likely biasing the population to those with less astigmatism than the general population. The exclusion of these lenses also limits the ability to analyze rotational stability of non-spherical lenses. Future studies could improve upon these limitations by including multiple refractive measurements or manual refraction at each follow-up, or multiple measurements after 6 weeks which can be averaged for reduced uncertainty in final stable post-operative refractive. Additional studies determining time to stabilization in patients with various comorbid ocular conditions, previous surgeries, alternative lenses (including torics, EDOF, three-piece, and others) or more eventful post-operative courses are also warranted.

In summary, these data suggest that refractive error can be effectively measured as early as one-week post-operatively in the vast majority of patients. Conservative clinicians, however, may choose to wait until other measures of stability including VA, CCT and ELP have stabilized – up to 4 weeks.

This study is one of several in recent years which suggest rapid stabilization of refraction following small-incision cataract surgery [8–10, 16]. In fact, a recent meta-analysis of the relevant literature showed that all published data suggests a more rapid course to refractive stability than the currently recommended 6 weeks, with study authors suggesting a range between one to 2 weeks post-operative for optimal time to correction [7]. Given these data, and the findings of this current report, these authors suggest that refractive correction may be offered to uncomplicated patients at 4 weeks, at which point all metrics measured in this study with potential to impact refractive error have stabilized. This will help to improve quality of life more rapidly post-operatively. However, additional efforts should be undertaken to identify patients that may be at increased risk of delayed refractive stability, such that clinicans may adjust their recommendations accordingly.

## Supplementary Information

**Additional file 1.**

## Data Availability

The data that support the findings of this study are available on request from the corresponding author, AK. The data are not publicly available due to information that could compromise the privacy of research participants.
