# Supplementary material for: Stabilization of refractive error and associated factors following small incision phacoemulsification cataract surgery
Source: BMC Ophthalmol. 2022 Jan 6;22:13. doi: 10.1186/s12886-021-02221-w (PMC8734338; doi:10.1186/s12886-021-02221-w)
Supplement: Supplementary file 1 — Additional file 1. [file 12886_2021_2221_MOESM1_ESM.pdf]

Supplemental Table 1:

	Week One	Week Two	Week Three	Week Four	Week Five	Week Six
**n=**	104	104	102	100	100	98
**Sphere (Dioptres; µ ± SD)**	-0.88 ± 0.77	-0.96 ± 0.78	-0.95 ± 0.60	-0.89 ± 0.64	-0.84 ± 0.65	-0.87 ± 0.72
**p-value (vs. Week 6)**	>0.99	0.47	0.48	0.99	>0.99	-
**Cylinder (Dioptres; µ ± SD)**	0.91 ± 0.64	0.90 ± 0.67	0.94 ± 0.63	0.97 ± 0.63	0.91 ± 0.60	0.94 ± 0.68
**p-value (vs. Week 6)**	0.98	0.90	>0.99	0.98	0.99	-
**Spherical Equivalent (Dioptres; µ ± SD)**	-0.43 ± 0.77	-0.53 ± 0.68	-0.49 ± 0.53	-0.42 ± 0.53	-0.39 ± 0.58	-0.41 ± 0.63
**p-value (vs. Week 6)**	>0.99	0.19	0.45	>0.99	>0.99	-
